# Supplementary material for: Correlated fragile site expression allows the identification of candidate fragile genes involved in immunity and associated with carcinogenesis
Source: BMC Bioinformatics. 2006 Sep 18;7:413. doi: 10.1186/1471-2105-7-413 (PMC1601973; doi:10.1186/1471-2105-7-413)
Supplement: Additional file 1 — Gene Ontology characterization of the connected component A1 at α = 1%. Gene Ontology characterization of the connected component A1 when the significance level for fragile site correlation is set to 1%. Significantly over-represented GO words are associated the full set of annotated genes. Genes' identifiers provided by the Hugo Gene Nomenclature Committee (Hugo ids) and genes' localization in fragile sites are reported. [file 1471-2105-7-413-S1.pdf]

Hugo id	*Fragile site*	*Hugo id*	*Fragile site*	*Hugo id*	*Fragile site*	*Hugo id*	*Fragile site*	*Hugo id*	*Fragile site*	*Hugo id*	*Fragile site*	*Hugo id*	*Fragile site*
**extracellular space (GO:0005615) :**													
CLCA3	FRA1D	AGL	FRA1E	SERPINC1	FRA1G	TNFSF18	FRA1G	TNFSF4	FRA1G	TNR	FRA1G	LIF	FRA22B
OSM	FRA22B	PLA2G3	FRA22B	TCN2	FRA22B	ADIPOQ	FRA3C	AHSG	FRA3C	KNG1	FRA3C	THPO	FRA3C
PLG	FRA6E	IFNA10	FRA9C	IFNA14	FRA9C	IFNA17	FRA9C	IFNA1	FRA9C	IFNA21	FRA9C	IFNA2	FRA9C
IFNA4	FRA9C	IFNA5	FRA9C	IFNA6	FRA9C	IFNA8	FRA9C	IFNB1	FRA9C	IFNK	FRA9C	IFNW1	FRA9C
NP_008831.2	FRA9C	NP_795372.1	FRA9C	KAL1	FRAXB								
**interferon-alpha/beta receptor binding (GO:0005132) :**													
IFNA10	FRA9C	IFNA17	FRA9C	IFNA1	FRA9C	IFNA2	FRA9C	IFNA4	FRA9C	IFNB1	FRA9C	IFNK	FRA9C
IFNW1	FRA9C												
**cytokine activity (G0:0005125) :**													
GLMN	FRA1D	TNFSF18	FRA1G	TNFSF4	FRA1G	LIF	FRA22B	OSM	FRA22B	THPO	FRA3C	IFNA10	FRA9C
IFNA14	FRA9C	IFNA17	FRA9C	IFNA1	FRA9C	IFNA21	FRA9C	IFNA2	FRA9C	IFNA4	FRA9C	IFNA5	FRA9C
IFNA6	FRA9C	IFNA8	FRA9C	IFNB1	FRA9C	IFNK	FRA9C	IFNW1	FRA9C	NP_008831.2	FRA9C	NP_795372.1	FRA9C
**hematopoietn/interferon-class (D200-domain) cytokine receptor (GO:0005126) :**													
LIF	FRA22B	OSM	FRA22B	IFNA10	FRA9C	IFNA14	FRA9C	IFNA17	FRA9C	IFNA1	FRA9C	IFNA21	FRA9C
IFNA2	FRA9C	IFNA4	FRA9C	IFNA5	FRA9C	IFNA6	FRA9C	IFNA8	FRA9C	IFNB1	FRA9C	IFNK	FRA9C
IFNW1	FRA9C	NP_008831.2	FRA9C	NP_795372.1	FRA9C								
**nuclear RNA export factor complex (G0:0042272) :**													
NXF2	FRAXC	NXF3	FRAXC										
**response to virus (GO:0009615) :**													
IFI44	FRA1C	IVNS1ABP	FRA1G	IFNA10	FRA9C	IFNA14	FRA9C	IFNA17	FRA9C	IFNA1	FRA9C	IFNA21	FRA9C
IFNA2	FRA9C	IFNA4	FRA9C	IFNA5	FRA9C	IFNA6	FRA9C	IFNA8	FRA9C	IFNB1	FRA9C	IFNK	FRA9C
IFNW1	FRA9C	NP_008831.2	FRA9C	NP_795372.1	FRA9C								
**protein binding (GO:0005515) :**													
NBEA	FRA13A	FHOD3	FRA18A	ANGPTL3	FRA1C	APG4C	FRA1C	DNAJB4	FRA1C	DNAJC6	FRA1C	DOCK7	FRA1C
FOXD3	FRA1C	IL12RB2	FRA1C	INADL	FRA1C	INSL5	FRA1C	ITGB3BP	FRA1C	LRRC7	FRA1C	NEGR1	FRA1C

Hugo id	Fragile site	Hugo id	Fragile site	Hugo id	Fragile site	Hugo id	Fragile site	Hugo id	Fragile site	Hugo id	Fragile site	Hugo id	Fragile site
**protein binding (GO:0005515) :**													
NP_060125.4	FRA1C	PAIRB_HUMAN	FRA1C	RABGGTB	FRA1C	RPE65	FRA1C	UBP33	FRA1C	BCL10	FRA1D	BRDT	FRA1D
BTBD8	FRA1D	CDC7	FRA1D	CYR61	FRA1D	DR1	FRA1D	EVI5	FRA1D	FNBP1L	FRA1D	GLMN	FRA1D
GTF2B	FRA1D	LRRC5	FRA1D	MTF2	FRA1D	RPL5	FRA1D	SEP15	FRA1D	SHLB1	FRA1D	SSX2IP	FRA1D
TMED5	FRA1D	DBT	FRA1E	SAS6	FRA1E	VCAM1	FRA1E	ANGPTL1	FRA1G	ASTN	FRA1G	DHX9	FRA1G
IVNS1ABP	FRA1G	KLHL20	FRA1G	LAMC1	FRA1G	LAMC2	FRA1G	NCF2	FRA1G	NIBA	FRA1G	NP_659471.1	FRA1G
NPHS2	FRA1G	RASAL2	FRA1G	RGL1	FRA1G	RGS16	FRA1G	SEN15	FRA1G	SERPINC1	FRA1G	STX6	FRA1G
TNFSF18	FRA1G	TNFSF4	FRA1G	TNR	FRA1G	ZBTB37	FRA1G	BTBD3	FRA20B	JAG1	FRA20B	MKKS	FRA20B
SNAP25	FRA20B	AP1B1	FRA22B	DRG1	FRA22B	EIF4ENIF1	FRA22B	EWSR1	FRA22B	LIF	FRA22B	LIMK2	FRA22B
NF2	FRA22B	OSM	FRA22B	SEC14L2	FRA22B	SEC14L3	FRA22B	SEC14L4	FRA22B	SMTN	FRA22B	TBC1D10A	FRA22B
ZNF278	FRA22B	SPTBN1	FRA2D	DNAJC10	FRA2H	FRZB	FRA2H	ITGAV	FRA2H	NCKAP1	FRA2H	PDE1A	FRA2H
ADIPOQ	FRA3C	AP2M1	FRA3C	BCL6	FRA3C	DNAJB11	FRA3C	DVL3	FRA3C	EIF4A2	FRA3C	EIF4G1	FRA3C
KLHL6	FRA3C	KNG1	FRA3C	MAP3K13	FRA3C	NP_060114.2	FRA3C	POLR2H	FRA3C	PSMD2	FRA3C	Q9BVH8	FRA3C
RFC4	FRA3C	SENP2	FRA3C	SST	FRA3C	THPO	FRA3C	CDH10	FRA5E	CDH12	FRA5E	CDH18	FRA5E
CDH9	FRA5E	PMCHL1	FRA5E	Q96RI3	FRA5E	PARK2	FRA6E	CAPZA2	FRA7G	CAV1	FRA7G	CAV2	FRA7G
CFTR	FRA7G	MET	FRA7G	TFEC	FRA7G	APTX	FRA9C	CDKN2A	FRA9C	DNAJA1	FRA9C	IFNA10	FRA9C
IFNA14	FRA9C	IFNA17	FRA9C	IFNA1	FRA9C	IFNA21	FRA9C	IFNA2	FRA9C	IFNA4	FRA9C	IFNA5	FRA9C
IFNA6	FRA9C	IFNA8	FRA9C	IFNB1	FRA9C	IFNK	FRA9C	IFNW1	FRA9C	KLHL9	FRA9C	NP_008831.2	FRA9C
NP_795372.1	FRA9C	TAF1L	FRA9C	KAL1	FRAXB	VCX3A	FRAXB	VCX	FRAXB	BTK	FRAXC	GPRASP2	FRAXC
NXF2	FRAXC	NXF2	FRAXC	NXF3	FRAXC	NXF5	FRAXC	SYTL4	FRAXC	TYBN	FRAXC		
