# Supplementary material for: Correlated fragile site expression allows the identification of candidate fragile genes involved in immunity and associated with carcinogenesis
Source: BMC Bioinformatics. 2006 Sep 18;7:413. doi: 10.1186/1471-2105-7-413 (PMC1601973; doi:10.1186/1471-2105-7-413)
Supplement: Additional file 2 — Gene Ontology characterization of the connected component B1 at α = 1% and α = 5%. Gene Ontology characterization of the connected component B1 when the significance level for fragile site correlation is set to 1% and to 5%. Significantly over-represented GO words are associated the full set of annotated genes. Genes' identifiers provided by the Hugo Gene Nomenclature Committee and genes' localizations in fragile sites are reported. [file 1471-2105-7-413-S2.pdf]

Hugo id	*Fragile site*	*Hugo id*	*Fragile site*	*Hugo id*	*Fragile site*	*Hugo id*	*Fragile site*	*Hugo id*	*Fragile site*	*Hugo id*	*Fragile site*	*Hugo id*	*Fragile site*
**carboxylesterase activity (GO:0004091) :**													
CTE1_HUMAN	FRA14C	PTE2A_HUMAN	FRA14C	PTE2B_HUMAN	FRA14C	CES2	FRA16C	NP_079198.2	FRA16C	NP_776176.2	FRA16C	ACHE	FRA7F
**serine esterase activity (GO:0004759) :**													
CTE1_HUMAN	FRA14C	PTE2A_HUMAN	FRA14C	PTE2B_HUMAN	FRA14C	CES2	FRA16C	NP_079198.2	FRA16C	NP_776176.2	FRA16C	ACHE	FRA7F
**xenobiotic metabolism (GO:0006805) :**													
NQO1	FRA16C	CYP3A43	FRA7F	CYP3A4	FRA7F	CYP3A5	FRA7F	CYP3A7	FRA7F				
