# Supplementary material for: Correlated fragile site expression allows the identification of candidate fragile genes involved in immunity and associated with carcinogenesis
Source: BMC Bioinformatics. 2006 Sep 18;7:413. doi: 10.1186/1471-2105-7-413 (PMC1601973; doi:10.1186/1471-2105-7-413)
Supplement: Additional file 4 — Gene Ontology characterization of the connected component ABC10at α = 10%. Gene Ontology characterization of the connected component ABC10 when the significance level for fragile site correlation is set to 10%. Significantly over-represented GO words are associated the full set of annotated genes. Genes' identifiers provided by the Hugo Gene Nomenclature Committee and genes' localizations in fragile sites are reported. [file 1471-2105-7-413-S4.pdf]

Hugo id	*Fragile site*	*Hugo id*	*Fragile site*	*Hugo id*	*Fragile site*	*Hugo id*	*Fragile site*	*Hugo id*	*Fragile site*	*Hugo id*	*Fragile site*	*Hugo id*	*Fragile site*
**cytokine activity (GO:0005125) :**													
GREM2	1q43	CKLF2	FRA16C	CKLF3	FRA16C	CKLF	FRA16C	CKLFSF4	FRA16C	GLMN	FRA1D	TNFSF18	FRA1G
TNFSF4	FRA1G	LIF	FRA22B	OSM	FRA22B	FAM3D	FRA3B	THPO	FRA3C	ARTS1	FRA5D	EPO	FRA7F
PBEF1	FRA7F	IFNA10	FRA9C	IFNA14	FRA9C	IFNA17	FRA9C	IFNA1	FRA9C	IFNA21	FRA9C	IFNA2	FRA9C
IFNA4	FRA9C	IFNA5	FRA9C	IFNA6	FRA9C	IFNA8	FRA9C	IFNB1	FRA9C	IFNK	FRA9C	IFNW1	FRA9C
NP_008831.2	FRA9C	NP_795372.1	FRA9C										
**hematopoietin/interferon-class (D200-domain) cytokine receptor (GO:0005126) :**													
LIF	FRA22B	OSM	FRA22B	ARTS1	FRA5D	EPO	FRA7F	IFNA10	FRA9C	IFNA14	FRA9C	IFNA17	FRA9C
IFNA1	FRA9C	IFNA21	FRA9C	IFNA2	FRA9C	IFNA4	FRA9C	IFNA5	FRA9C	IFNA6	FRA9C	IFNA8	FRA9C
IFNB1	FRA9C	IFNK	FRA9C	IFNW1	FRA9C	NP_008831.2	FRA9C	NP_795372.1	FRA9C				
**interferon-alpha/beta receptor binding (GO:0005132) :**													
IFNA10	FRA9C	IFNA17	FRA9C	IFNA1	FRA9C	IFNA2	FRA9C	IFNA4	FRA9C	IFNB1	FRA9C	IFNK	FRA9C
IFNW1	FRA9C												
**protein carrier activity (GO:0008320):**													
TMED8	FRA14C	TMED6	FRA16C	TMED5	FRA1D	SEC14L2	FRA22B	SEC14L3	FRA22B	SEC14L4	FRA22B	AZGP1	FRA7F
**response to virus (GO:0009615) :**													
IFI44	FRA1C	IVNS1ABP	FRA1G	IFNA10	FRA9C	IFNA14	FRA9C	IFNA17	FRA9C	IFNA1	FRA9C	IFNA21	FRA9C
IFNA2	FRA9C	IFNA4	FRA9C	IFNA5	FRA9C	IFNA6	FRA9C	IFNA8	FRA9C	IFNB1	FRA9C	IFNK	FRA9C
IFNW1	FRA9C	NP_008831.2	FRA9C	NP_795372.1	FRA9C								
