# Supplementary material for: Correlated fragile site expression allows the identification of candidate fragile genes involved in immunity and associated with carcinogenesis
Source: BMC Bioinformatics. 2006 Sep 18;7:413. doi: 10.1186/1471-2105-7-413 (PMC1601973; doi:10.1186/1471-2105-7-413)
Supplement: Additional file 5 — Gene Ontology characterization of the connected component D10 at α = 10%. Gene Ontology characterization of the connected component D10 when the significance level for fragile site correlation is set to 10%. Significantly over-represented GO words are associated the full set of annotated genes. Genes' identifiers provided by the Hugo Gene Nomenclature Committee and genes' localizations in fragile sites are reported. [file 1471-2105-7-413-S5.pdf]

Hugo id	***Fragile site***	***Hugo id***	***Fragile site***	***Hugo id***	***Fragile site***	***Hugo id***	***Fragile site***	***Hugo id***	***Fragile site***	***Hugo id***	***Fragile site***	***Hugo id***	***Fragile site***
**nucleic acid binding (GO:0003676) :**													
CREB5	7p15	EVX1	7p15	HNRPA2B1	7p15	HOXA1	7p15	HOXA10	7p15	HOXA11	7p15	HOXA13	7p15
HOXA2	7p15	HOXA3	7p15	HOXA4	7p15	HOXA5	7p15	HOXA6	7p15	HOXA7	7p15	HOXA9	7p15
NFE2L3	7p15	JAZF1_HUMAN	7p15	NP_006538.2	7p15	NUPL2	7p15	OSBPL3	7p15	SP4	7p15	STK31	7p15
TAX1BP1	7p15	TRA2A_HUMAN	7p15	XP_374414.2	7p15	DDX46	FRA5C	H2AFY	FRA5C	HNRPA0	FRA5C	NEUROG1	FRA5C
NP_055238.1	FRA5C	PITX1	FRA5C	SMAD5	FRA5C	TCF7	FRA5C	ZCCHC10	FRA5C				
**transcription regulator activity (G0:0030528) :**													
CREB5	7p15	EVX1	7p15	HOXA1	7p15	HOXA10	7p15	HOXA11	7p15	HOXA13	7p15	HOXA2	7p15
HOXA3	7p15	HOXA4	7p15	HOXA5	7p15	HOXA6	7p15	HOXA7	7p15	HOXA9	7p15	NFE2L3	7p15
SP4	7p15	NEUROG1	FRA5C	NP_055238.1	FRA5C	PITX1	FRA5C	SMAD5	FRA5C	TCF7	FRA5C		
**transcription factor activity (GO:0003700) :**													
CREB5	7p15	EVX1	7p15	HOXA1	7p15	HOXA10	7p15	HOXA11	7p15	HOXA13	7p15	HOXA2	7p15
HOXA3	7p15	HOXA4	7p15	HOXA5	7p15	HOXA6	7p15	HOXA7	7p15	HOXA9	7p15	NFE2L3	7p15
NEUROG1	FRA5C	NP_055238.1	FRA5C	PITX1	FRA5C	SMAD5	FRA5C						
