# Supplementary material for: Correlated fragile site expression allows the identification of candidate fragile genes involved in immunity and associated with carcinogenesis
Source: BMC Bioinformatics. 2006 Sep 18;7:413. doi: 10.1186/1471-2105-7-413 (PMC1601973; doi:10.1186/1471-2105-7-413)
Supplement: Additional file 6 — Full list of triangles detected at α = 1%. The full list of triangles detectable at the significance level for fragile site correlated expression set to α = 1%. [file 1471-2105-7-413-S6.pdf]

FRA1E	FRA1C	FRA13A
FRA1E	FRA1C	FRA22B
FRA1C	FRA7G	FRA2H
FRA11D	FRA1C	FRA2H
FRA1C	FRA13A	FRA2H
FRA1C	FRA2H	FRA18A
FRA1C	FRA2H	FRA20B
FRA1C	FRA2H	FRA22B
FRA1C	FRA3C	FRA22B
FRA11D	FRA5E	FRA1C
FRA5E	FRA1C	FRA20B
FRA1C	FRA7G	FRA20B
FRA1C	FRA7G	FRA22B
FRA11D	FRA1C	FRA20B
FRA11D	FRA1C	FRA22B
FRA1C	FRA13A	FRA20B
FRA1C	FRA13A	FRA22B
FRA1C	FRA22B	FRA20B
FRA1C	FRAXB	FRA22B
FRA1E	FRA13A	FRA22B
FRA7G	FRA2H	FRA20B
FRA7G	FRA2H	FRA22B
FRA11D	FRA2H	FRA20B
FRA11D	FRA2H	FRA22B
FRA13A	FRA2H	FRA20B
FRA13A	FRA2H	FRA22B
FRA2H	FRA22B	FRA20B
FRA1G	FRA3C	FRA22B
FRA11D	FRA5E	FRA20B
FRA7G	FRA22B	FRA20B
FRA1G	FRA7G	FRA20B
FRA1G	FRA7G	FRA22B
FRA11D	FRA22B	FRA20B
FRA13A	FRA22B	FRA20B
FRA1G	FRA22B	FRA20B
FRA1D	FRAXB	FRA22B
