# Supplementary material for: Correlated fragile site expression allows the identification of candidate fragile genes involved in immunity and associated with carcinogenesis
Source: BMC Bioinformatics. 2006 Sep 18;7:413. doi: 10.1186/1471-2105-7-413 (PMC1601973; doi:10.1186/1471-2105-7-413)
Supplement: Additional file 8 — Cytogenetic position of fragile sites. Cytogenetic position of fragile sites is reported in the first column; when cytogenetic regions where fragility has been observed include annotated fragile sites, we specify annotated fragile sites along with their genomic position. Fragile sites' genomic lengths and overall breakage occurrences are provided as well. [file 1471-2105-7-413-S8.pdf]

Fragile site	Gene Bank id	Gene Bank position	Genomic length (Mbp)	Breakage number
1p36	FRA1A	1p36	27,6	76
1p32	FRA1B	1p32	9,7	87
1p31	FRA1C	1p31	4,0	154
1p22	FRA1D	1p22	9,9	325
1p21	FRA1E	1p21.2	2,5	46
1p13		1p13	10,6	28
1q21	FRA1F	1q21	9,8	24
1q22		1q22	2,0	23
1q24		1q24	7,4	22
1q25	FRA1G	1q25	3,1	178
1q31	FRA1K	1q31	13,5	25
1q32		1q32	14,5	56
1q42	FRA1H	1q42.1	7,2	41
1q43		1q43	6,6	26
1q44	FRA1I	1q44	6,0	357
2p24	FRA2C	2p24.2	2,5	31
2p23		2p23	8,0	49
2p21		2p21	6,0	21
2p16	FRA2D	2p16.2	2,1	30
2p15		2p15	2,8	12
2p13	FRA2E	2p13	6,5	50
2q21	FRA2F	2q21.3	2,5	64
2q23		2q23	6,2	18
2q31	FRA2G	2q31	13,2	63
2q32	FRA2H	2q32.1	14,5	134
2q33	FRA2I	2q33	11,6	135
2q35		2q35	6,2	38
2q36		2q36	9,4	21
2q37	FRA2J	2q37.3	5,9	102
3p26		3p26	8,2	23
3p25		3p25	8,2	37

Fragile site	Gene Bank id	Gene Bank position	Genomic length (Mbp)	Breakage number
3p24	FRA3A	3p24.2	3,3	53
3p23		3p23	2,8	12
3p21		3p21	10,8	65
3p14	FRA3B	3p14.2	5,2	1447
3p13		3p13	4,3	19
3p12		3p12	13,0	28
3q21		3q21	9,4	25
3q25	FRA3D	3q25	11,7	41
3q27	FRA3C	3q27	5,3	176
3q29		3q29	5,5	16
4p16	FRA4A	4p16.1	5,1	21
4q25		4q25	6,4	28
4q27	FRA4E	4q27	3,2	10
4q31	FRA4C	4q31.1	2,0	62
4q33		4q33	1,8	9
5p15		5p15	18,4	33
5p14	FRA5E	5p14	10,8	50
5p13	FRA5A	5p13	13,1	25
5q15	FRA5D	5q15	5,4	47
5q21	FRA5F	5q21	12,3	32
5q31	FRA5C	5q31.1	5,0	274
6p25	FRA6B	6p25.1	7,0	113
6p23		6p23	1,8	23
6q15	FRA6G	6q15	4,6	14
6q21	FRA6F	6q21	9,4	47
6q23		6q23	8,7	29
6q25		6q25	11,9	130
6q26	FRA6E	6q26	3,5	160
6q27		6q27	6,5	40
7p22	FRA7B	7p22	7,0	179
7p15		7p15	9,5	23

Fragile site	Gene Bank id	Gene Bank position	Genomic length (Mbp)	Breakage number
7p14		7p14	2,3	49
7p13	FRA7D	7p13	4,0	26
7q21	FRA7E	7q21.2	8,7	24
7q22	FRA7F	7q22	9,3	340
7q31	FRA7G	7q31.2	2,8	141
7q32	FRA7H	7q32.3	2,1	806
8q22	FRA8B	8q22.1	5,7	77
8q24	FRA8D	8q24.3	9,6	35
9p23		9p23	5,1	18
9p21	FRA9C	9p21	13,3	36
9q12	FRA9F	9q12	8,3	18
9q22	FRA9D	9q22.1	1,4	91
9q32	FRA9E	9q32	2,8	58
9q34		9q34	11,1	23
10p13		10p13	5,0	16
10q22	FRA10D	10q22.1	4,3	22
10q24		10q24	8,7	36
10q25		10q25	3,1	21
10q26	FRA10F	10q26.1	8,7	61
11p14	FRA11D	11p14.2	1,2	74
11p13	FRA11E	11p13	5,4	47
11q14	FRA11F	11q14.2	2,5	16
12p12		12p12	11,7	23
12q21	FRA12B	12q21.3	4,3	30
12q22		12q22	3,5	15
13q13	FRA13A	13q13.2	1,6	176
13q14		13q14	15,2	66
13q21	FRA13C	13q21.2	2,7	28
13q22		13q22	5,8	23
13q32	FRA13D	13q32	6,7	36
14q13		14q13	4,5	30

Fragile site	Gene Bank id	Gene Bank position	Genomic length (Mbp)	Breakage number
14q21		14q21	13,2	38
14q22		14q22	7,1	24
14q23	FRA14B	14q23	9,8	30
14q24	FRA14C	14q24	2,3	453
15q15		15q15	4,7	53
15q22	FRA15A	15q22	8,3	26
16q13		16q13	1,4	6
16q21		16q21	7,2	43
16q22	FRA16C	16q22.1	5,5	779
16q23	FRA16D	16q23.2	2,1	421
16q24		16q24	6,1	88
17q23	FRA17B	17q23.1	0,3	35
18p11		18p11	16,1	30
18q12	FRA18A	18q12.2	4,5	54
18q21	FRA18B	18q21.3	7,3	44
20p13		20p13	5,0	14
20p12	FRA20B	20p12.2	2,9	59
22q12	FRA22B	22q12.2	2,6	118
22q13		22q13	13,7	65
Xp22	FRAXB	Xp22.31	3,5	355
Xq22	FRAXC	Xq22.1	4,2	182
Xq26		Xq26	8,0	28
Xq27	FRAXD	Xq27.2	1,8	18
